# HEPATIC ENCEPHALOPATHY IN ALCOHOL-RELATED CIRRHOSIS:A SYSTEMATIC REVIEW OF CLINICAL CHARACTERISTICS, THERAPEUTIC STRATEGIES, AND PROGNOSTIC OUTCOMES

**DOI:** 10.1590/S0004-2803.24612026-066

**Published:** 2026-10-30

**Authors:** Gilberto JARAMILLO-TRUJILLO, Juan Sebastián FRÍAS-ORDOÑEZ, Hernando MARULANDA-FERNANDEZ, William OTERO-REGINO, Jorge Armando MEJÍA-TOBAR

**Affiliations:** 1 National University of Colombia, Gastroenterology, Bogotá, Colombia.; 2 San Rafael Hospital of Facatativá, Gastroenterology, Cundinamarca, Colombia.; 3 International Hospital of Colombia, Gastroenterology, Bucaramanga, Colombia.; 4 Cardiovascular Foundation of Colombia, Research Group in Medical and Surgical Specialties, Floridablanca, Colombia.; 5 Central Police Hospital, Gastroenterology and Digestive Endoscopy, Bogotá, Colombia.; 6 Occidente de Kennedy Hospital, Gastroenterology and Digestive Endoscopy, Bogotá, Colombia.; 7 Santa Clara Hospital, Gastroenterology and Digestive Endoscopy, Bogotá, Colombia.; 8 Center for Gastroenterology and Endoscopy, Bogotá, Colombia.; 9 University Foundation of Health Sciences - San José Hospital, Clinical Toxicology, Bogotá, Colombia.; 10 Mental Health Foundation of Valle, Jamundí, Colombia.

**Keywords:** Hepatic encephalopathy, liver cirrhosis, alcohol-related liver disease, alcohol-related disorders, rifaximin, Encefalopatia hepática, cirrose hepática, doença hepática relacionada ao álcool, transtornos relacionados ao álcool, rifaximina

## Abstract

**Background:**

Hepatic encephalopathy (HE) is a major complication of cirrhosis; however, its characterization in alcohol-related cirrhosis (ARC) remains limited because most available evidence derives from mixed-etiology cohorts.

**Methods:**

A systematic review was conducted according to PRISMA 2020 guidelines. PubMed, EMBASE, and LILACS were searched through April 2026. Randomized controlled trials and cohort studies involving adults with ARC or documented alcohol consumption reporting HE-related outcomes were included. Risk of bias was assessed using the Cochrane RoB 2.0 tool and the Newcastle-Ottawa Scale, while certainty of evidence was evaluated using the GRADE framework. Due to substantial heterogeneity, a narrative synthesis was performed.

**Results:**

Fourteen studies involving 6,185 participants were included. HE was associated with a hospital prevalence of 18.5% and a 90-day mortality of 12.1%. Observational evidence suggested that HE was associated with increased mortality (HR 3.1 at 90 days; HR 2.9 at 1 year), while active alcohol consumption was identified as a potential adverse prognostic factor (HR 2.5). For secondary prophylaxis, rifaximin was associated with reduced recurrence (HR 0.42), predominantly as add-on therapy to lactulose. Lactulose also reduced recurrence compared with placebo. In acute HE, polyethylene glycol demonstrated faster clinical improvement than lactulose, although evidence was limited.

**Conclusion:**

Available evidence suggests that HE in alcohol-related cirrhosis may represent a clinically relevant subgroup with potentially distinct prognostic characteristics. However, current evidence remains indirect, heterogeneous, and largely derived from mixed-etiology populations, precluding definitive conclusions regarding a specific alcohol-related HE phenotype. Dedicated prospective studies are needed.

## HIGHLIGHTS

• Fourteen studies including 6,185 patients were systematically reviewed.

• Hepatic encephalopathy was associated with increased short- and long-term mortality.

• Active alcohol consumption was associated with worse prognosis.

• Rifaximin and lactulose reduced hepatic encephalopathy recurrence.

• Alcohol-specific evidence remains limited and heterogeneous.

## RESUMO

**Contexto:**

A encefalopatia hepática (EH) é uma importante complicação da cirrose; entretanto, sua caracterização na cirrose relacionada ao álcool (CRA) permanece limitada, uma vez que a maior parte das evidências disponíveis deriva de coortes com etiologias mistas.

**Métodos:**

Foi realizada uma revisão sistemática de acordo com as diretrizes PRISMA 2020. As bases PubMed, EMBASE e LILACS foram pesquisadas até abril de 2026. Foram incluídos ensaios clínicos randomizados e estudos de coorte envolvendo adultos com CRA ou consumo de álcool documentado que relatassem desfechos relacionados à EH. O risco de viés foi avaliado utilizando a ferramenta Cochrane RoB 2.0 e a Escala Newcastle-Ottawa, enquanto a certeza da evidência foi avaliada por meio da abordagem GRADE. Devido à heterogeneidade substancial entre os estudos, foi realizada uma síntese narrativa.

**Resultados:**

Foram incluídos quatorze estudos envolvendo 6.185 participantes. A EH esteve associada a uma prevalência hospitalar de 18,5% e a uma mortalidade em 90 dias de 12,1%. Evidências observacionais sugeriram que a EH esteve associada ao aumento da mortalidade (HR 3,1 em 90 dias; HR 2,9 em 1 ano), enquanto o consumo ativo de álcool foi identificado como um potencial fator prognóstico adverso (HR 2,5). Na profilaxia secundária, a rifaximina esteve associada à redução da recorrência (HR 0,42), predominantemente como terapia adjuvante à lactulose. A lactulose também reduziu a recorrência em comparação ao placebo. Na EH aguda, o polietilenoglicol demonstrou melhora clínica mais rápida do que a lactulose, embora as evidências sejam limitadas.

**Conclusão:**

As evidências disponíveis sugerem que a EH na cirrose relacionada ao álcool pode representar um subgrupo clinicamente relevante, com características prognósticas potencialmente distintas. No entanto, as evidências atuais permanecem indiretas, heterogêneas e amplamente derivadas de populações com etiologias mistas, impedindo conclusões definitivas sobre a existência de um fenótipo específico de EH relacionada ao álcool. São necessários estudos prospectivos dedicados.

## INTRODUCTION

Hepatic encephalopathy (HE) is one of the most important complications of cirrhosis and is associated with impaired quality of life, reduced functional status, increased healthcare utilization, and decreased survival^1^ ^,^ ^2^. Overt HE develops in approximately 30-45% of patients with cirrhosis during the course of their disease, while minimal or covert forms are even more common^3^ ^,^ ^4^. Despite advances in diagnosis and management, HE remains a major cause of hospitalization, recurrent decompensation, and mortality among patients with advanced liver disease^1^ ^-^ ^4^.

Alcohol-related cirrhosis represents a major cause of liver-related morbidity and mortality worldwide and remains one of the leading indications for hospitalization and liver transplantation in many regions^5^ ^,^ ^6^. The coexistence of advanced liver disease and alcohol use disorder may influence the clinical course of HE through effects on disease progression, treatment adherence, and long-term outcomes. Although several experimental studies have suggested potential mechanisms linking alcohol exposure to neuroinflammation and neurotoxicity, the clinical implications of these observations remain incompletely understood^7^ ^,^ ^8^.

Despite the clinical relevance of alcohol-related cirrhosis, most evidence regarding the diagnosis, treatment, and prognosis of HE derives from mixed-etiology cirrhotic populations. Alcohol-related cirrhosis is rarely analyzed as a distinct subgroup, and ongoing alcohol consumption is infrequently reported or incorporated into outcome analyses. Consequently, current clinical guidelines provide recommendations for HE in cirrhosis but do not specifically address alcohol-related cirrhosis^1^ ^,^ ^8^. This evidence gap limits the ability to determine whether patients with alcohol-related cirrhosis exhibit distinct clinical, prognostic, or therapeutic characteristics.

The rationale for this review stems from the observation that alcohol-related cirrhosis is frequently included within mixed-etiology cohorts, limiting the ability to determine whether HE in this population exhibits specific clinical, prognostic, or therapeutic characteristics. Experimental and clinical studies have suggested potential mechanisms through which alcohol exposure may influence the development and outcomes of HE; however, the extent to which these observations translate into meaningful differences in clinical practice remains uncertain.

Accordingly, the aim of this systematic review was to synthesize the available evidence regarding the prevalence, clinical characteristics, therapeutic strategies, and prognostic outcomes of HE in patients with alcohol-related cirrhosis. By summarizing current evidence and identifying important knowledge gaps, this review seeks to inform future research and improve understanding of this clinically relevant population.

## METHODS

### Study design and research question

A systematic review was conducted to identify, critically appraise, and synthesize the available evidence on HE in adult patients with alcohol-related cirrhosis or documented alcohol consumption. The research question was structured according to the PICO framework: Population (adults with alcohol-related cirrhosis or documented alcohol use); Intervention/Exposure (pharmacological treatments, prognostic factors, and biomarkers); Comparator (placebo, active comparator, or absence of HE); and Outcomes (HE recurrence, clinical resolution, mortality, and hospital readmissions).

### Eligibility criteria

Studies were eligible if they included adult patients (≥18 years) with cirrhosis defined by clinical, biochemical, imaging, or histological criteria, and met the following conditions: (a) alcohol-related etiology, explicitly reported alcohol-related liver disease (ALD), or documented alcohol consumption; and (b) reporting of outcomes related to HE, including recurrence, hospitalization, or mortality. Randomized controlled trials and observational cohort studies (prospective or retrospective) were included.

Exclusion criteria comprised pediatric populations, studies in which HE was not associated with cirrhosis (e.g., isolated acute liver failure), de novo acute liver failure, and studies lacking specific HE-related outcomes. For acute treatment studies, HE diagnosis was required to be based on standardized criteria (West Haven classification, Conn score, or HESA), with appropriate exclusion of alternative causes of altered mental status.

### Search strategy

A comprehensive electronic search was performed in PubMed, EMBASE, and LILACS from database inception through April 2026. The strategy combined controlled vocabulary (MeSH, Emtree, DeCS) and free-text terms related to “hepatic encephalopathy,” “liver cirrhosis”, “alcohol”, “alcohol-related liver disease”, “alcoholic liver disease”, and “alcohol use disorder”, including Spanish equivalents. The complete electronic search strategies for PubMed, EMBASE, and LILACS are provided in SUPPLEMENTARY MATERIAL 1.

**SUPPLEMENTARY APPENDIX 1.** Complete Search Strategies

SUPPLEMENTARY MATERIAL

Database: PubMed
Search date: April 15, 2026
((“hepatic encephalopathy”[MeSH Terms] OR “hepatic encephalopathy”[Title/Abstract] OR “hepatic encephalopathy syndrome”[Title/Abstract])
AND
(“liver cirrhosis”[MeSH Terms] OR cirrhosis[Title/Abstract] OR “hepatic cirrhosis”[Title/Abstract])
AND
(“alcohol-related liver disease”[Title/Abstract] OR “alcoholic liver disease”[Title/Abstract] OR “alcohol-related cirrhosis”[Title/Abstract] OR “alcoholic cirrhosis”[Title/Abstract] OR alcohol[Title/Abstract] OR “alcohol use disorder”[Title/Abstract] OR AUD[Title/Abstract]))

Database: EMBASE
Search date: April 15, 2026
(‘hepatic encephalopathy’/exp OR ‘hepatic encephalopathy’,ab)
AND
(‘liver cirrhosis’/exp OR cirrhosis,ab OR ‘hepatic cirrhosis’,ab)
AND
(‘alcohol related liver disease’,ab
OR ‘alcoholic liver disease’,ab
OR ‘alcoholic cirrhosis’,ab
OR ‘alcohol related cirrhosis’,ab
OR alcohol,ab
OR ‘alcohol use disorder’,ab)

Database: LILACS
Search date: April 15, 2026
((mh:(Encefalopatía Hepática) OR “encefalopatia hepática” OR “hepatic encephalopathy”))
AND
((mh:(Cirrosis Hepática) OR “cirrose hepática” OR “liver cirrhosis”))
AND
((“doença hepática relacionada ao álcool”)
OR
(“enfermedad hepática relacionada con el alcohol”)
OR
(“alcoholic cirrhosis”)
OR
(“alcohol-related liver disease”)
OR
(“alcohol use disorder”))

To ensure completeness, the search was supplemented by manual review of reference lists from included studies and relevant reviews, as well as grey literature searches in ClinicalTrials.gov, the WHO International Clinical Trials Registry Platform (ICTRP), and institutional repositories. No language restrictions were applied. The review protocol was not prospectively registered; however, the study was conducted in accordance with predefined methodological criteria and PRISMA 2020 guidelines.

### Study selection

After removal of duplicates, studies were screened in two stages: (1) title and abstract screening, and (2) full-text assessment of potentially eligible articles. Screening was conducted independently by multiple reviewers at each stage, with discrepancies resolved through consensus, ensuring methodological rigor and reproducibility.

### Data extraction

Data were extracted using a standardized form capturing: study characteristics (author, year, country), design, sample size, diagnostic criteria for cirrhosis and HE, severity of liver disease (Child-Pugh and MELD scores), assessment of alcohol exposure, interventions or exposures evaluated, follow-up duration, clinical outcomes, and key quantitative results. When multiple publications referred to the same study population, data were consolidated to avoid duplication.

### Risk of bias assessment

Risk of bias was evaluated using validated tools according to study design: the Cochrane Risk of Bias 2.0 tool for randomized controlled trials and the Newcastle-Ottawa Scale (NOS) for observational studies. Particular attention was given to missing data, selective outcome reporting, and heterogeneity in HE definitions.

### Certainty of evidence assessment

The certainty of evidence for the main outcomes was assessed using the Grading of Recommendations Assessment, Development and Evaluation (GRADE) approach. Certainty was rated as high, moderate, low, or very low according to study design and the presence of risk of bias, inconsistency, indirectness, imprecision, and publication bias. Randomized controlled trials initially started as high-certainty evidence, whereas observational studies initially started as low-certainty evidence. Downgrading was applied when limitations were identified, particularly indirectness due to reliance on mixed-etiology cirrhotic populations, inconsistency across study definitions, and imprecision related to the limited number of studies and events.

### Data synthesis

Given the substantial clinical, methodological, and statistical heterogeneity across studies-including variability in populations, definitions of HE, alcohol exposure assessment, interventions, and outcome measures-a quantitative meta-analysis was not performed. Instead, a structured qualitative synthesis was undertaken.

Results were organized into clinically relevant domains: (1) acute inpatient management, (2) secondary prophylaxis and prevention of recurrence, (3) primary prophylaxis, and (4) prognostic factors and biomarkers. Within each domain, consistency of findings, direction and magnitude of effects, and methodological limitations were systematically evaluated to provide a robust and clinically meaningful interpretation.

## RESULTS

### Study selection

The systematic search across the three databases identified a total of 1,298 records. After removal of 260 duplicates, 1,038 records underwent title and abstract screening, of which 1,011 were excluded for not meeting eligibility criteria. Twenty-seven records were sought for full-text retrieval; one could not be accessed. Of the 26 articles assessed in full, 12 were excluded (7 due to duplicate reporting of already included studies and 5 for not meeting population or outcome criteria). Ultimately, 14 studies were included in the qualitative synthesis.

The study selection process is summarized in Figure 1 (PRISMA 2020 flow diagram). A total of 1,298 records were identified through database searches. After removal of 260 duplicates, 1,038 records were screened. Twenty-seven full-text articles were sought for retrieval; one was not available, leaving 26 full-text articles assessed for eligibility. Following full-text review, 12 articles were excluded (7 duplicate reports and 5 not meeting population or outcome criteria). Ultimately, 14 studies met the eligibility criteria and were included in the qualitative synthesis.

**FIGURE 1:**
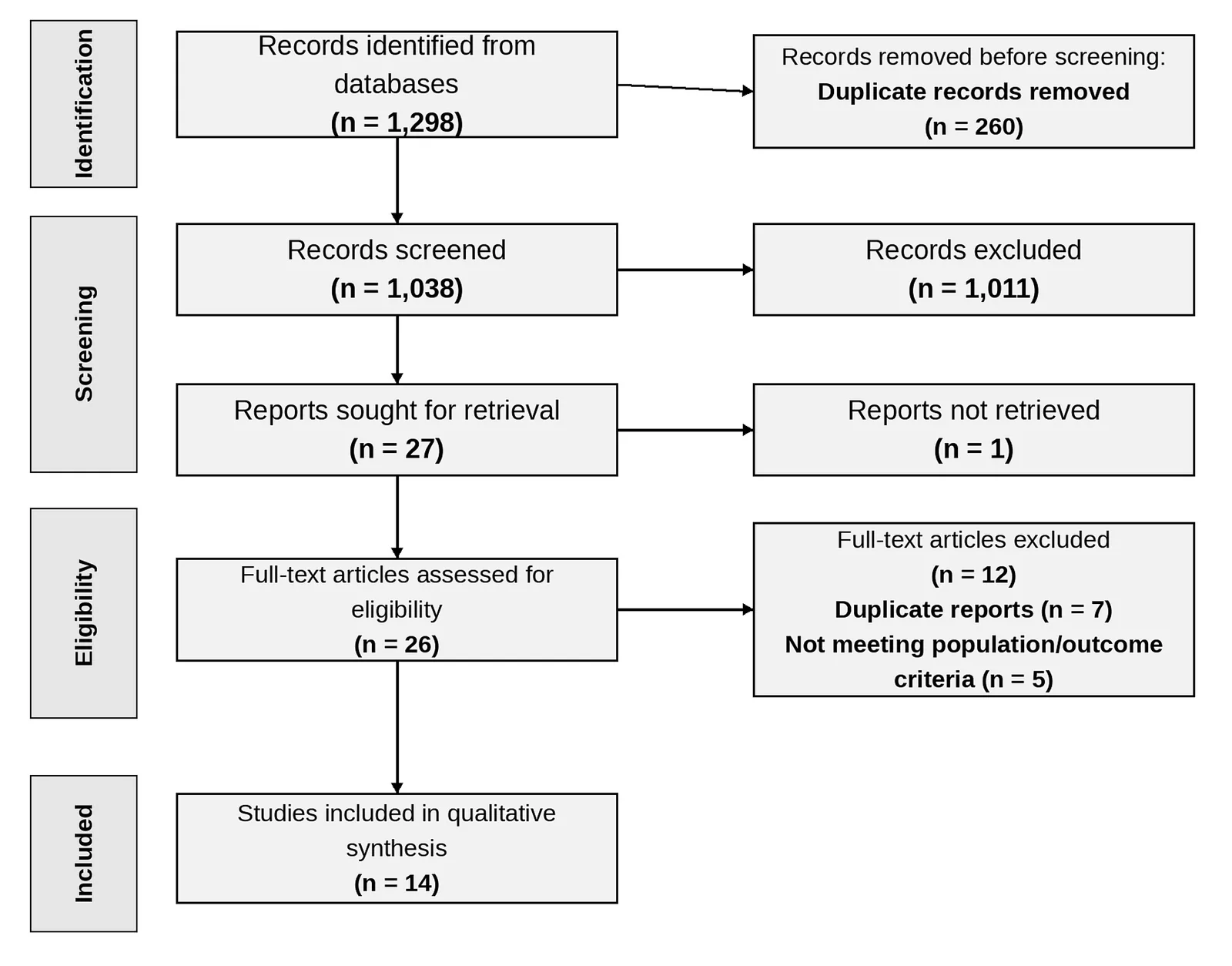
PRISMA Flow Diagram. Flow diagram of study selection according to the Preferred Reporting Items for Systematic Reviews and Meta-Analyses (PRISMA) 2020 guidelines. A total of 1,298 records were identified through database searching. After removal of duplicates (n=260), 1,038 records were screened, of which 1,011 were excluded based on title and abstract. Twenty-seven articles were sought for full-text retrieval; one was not available. Of the 26 full-text articles reviewed, 12 were excluded (7 duplicate reports and 5 not meeting inclusion criteria). Fourteen studies were included in the final qualitative synthesis.

### Study characteristics

The 14 included studies comprised a total of 6,185 participants, with sample sizes ranging from 20 to 2,603 individuals. Study designs included nine randomized controlled trials and five observational cohort studies (three retrospective and two prospective). The geographic distribution was diverse, including studies from the United States (n=3), India (n=4), Italy (n=2), France (n=1), Spain (n=1), Sweden (n=1), South Korea (n=1), and one international multicenter study. Detailed characteristics are provided in Table 1.

**TABLE 1:** Summary of the main characteristics of the studies included in the systematic review.

Author (Year)	Country	Study Design	Sample Size	Diagnostic Criteria (Cirrhosis/HE)	Severity (Child-Pugh/MELD)	Alcohol Exposure (Definition/Assessment)	Interventions/Exposures	Follow-up
Bass et al., 2010^9^	Multicenter (USA and others)	RCT, double-blind, placebo-controlled	299 (rifaximin 140; placebo 159)	Cirrhosis; patients in remission from recurrent HE; clinical assessment (West Haven/Conn typical; details NR)	NR	Etiology not stratified; alcohol involvement NR	Rifaximin 550 mg BID vs placebo; frequent concomitant lactulose	6 months
Rahimi et al., 2014^12^	USA	RCT	50 (PEG 25; lactulose 25)	Cirrhosis with altered mental status; HE defined clinically; HESA for severity	Mean MELD 17; mean CTP 10	ALD in 38% overall	PEG 4 L vs standard lactulose (acute setting)	24 h (primary); until resolution
Sharma et al., 2009^10^	India	RCT (open-label)	140	Cirrhosis post-HE episode; psychometry, CFF, ammonia; outcome: OHE recurrence	NR	Alcohol etiology 39%	Lactulose vs placebo (secondary prophylaxis)	Median 14 months
Agrawal et al., 2012^11^	India	RCT (open-label)	197	Cirrhosis post-HE; intervention-based definition	NR	Alcohol etiology likely (NR)	Lactulose vs probiotics vs no therapy	NR
Lunia et al., 2014^14^	India	RCT	160	Cirrhosis without OHE; PHES/CFF; SIBO/OCTT assessed	CTP A-C distribution	Alcohol etiology NR	Probiotics vs control (primary prophylaxis)	Up to 6 months
Sharma et al., 2008^15^	India	RCT (open-label)	105 (MHE subgroup)	MHE defined by psychometry and/or P300	Child A-C	Alcohol etiology NR	Lactulose vs probiotics vs combination	1 month
Paik et al., 2005^13^	South Korea	RCT	54	Cirrhosis with HE (definition NR)	NR	Alcohol etiology NR	Rifaximin vs lactulose	NR
Vierling et al., 2014 (HALT-HE)^16^	Multicenter	Phase II RCT, double-blind	178	Cirrhosis with ≥2 HE events in prior 6 months	NR	Alcohol etiology NR	Glycerol phenylbutyrate vs placebo	NR
Bajaj et al., 2017^17^	USA	Pilot RCT	20	Cirrhosis with recurrent HE; SOC-based	NR	Alcohol etiology NR	FMT vs SOC	5 months
Ventura-Cots et al., 2021^18^	Spain	RCT, double-blind	82	Acute HE ≥ grade II	NR	ALD subgroup NR	Albumin vs placebo	90 days
Nardelli et al., 2022^19^	Italy	Retrospective cohort	1,429	Cirrhosis (clinical); HE by West Haven	MELD median 16; Child B-C predominant	Alcohol-related cirrhosis	Prognostic factors (AKI, bleeding, infection, etc.)	90 days
Italian multicenter cohort, 2020^19^	Italy	Prospective cohort	368	Decompensated cirrhosis; HE at admission	MELD higher in HE	Alcohol stratified	HE vs no HE (mortality outcomes)	90 days; 12 months
Boeckmans et al., 2025^21^	Sweden	Population-based cohort	2,603	Registry-based cirrhosis (ICD codes)	NR	ALD vs MASLD classification	Etiology comparison	Median 25 months
Elkrief et al., 2023^22^	France	Cohort	500	Compensated ALD cirrhosis; liver-related events	Child A	Active vs inactive alcohol use	K18, lEV biomarkers	2 years

RCT: randomized controlled trial. HE: hepatic encephalopathy. MHE: minimal hepatic encephalopathy. OHE: overt hepatic encephalopathy. CTP: child-turcotte-pugh. MELD: model for end-stage liver disease. HESA: hepatic encephalopathy scoring algorithm. CFF: critical flicker frequency. SIBO: small intestinal bacterial overgrowth. OCTT: orocecal transit time. SOC: standard of care. NR: not reported.

The definition of alcohol-related cirrhosis varied across studies. Some studies used alcohol-related cirrhosis as an inclusion criterion, whereas others analyzed alcohol-related liver disease (ALD) as a stratified subgroup, classified patients based on registry-derived etiologies (for example, ALD vs MASLD), or incorporated active alcohol consumption as an exposure variable. This heterogeneity in etiological classification represents an inherent limitation of the evidence base and is addressed in the Discussion.

### Distribution of included studies by research domain

The 14 included studies were grouped according to their primary research objective. Nine randomized controlled trials evaluated therapeutic interventions for hepatic encephalopathy, including secondary prophylaxis (n=4), acute treatment (n=2), primary prophylaxis (n=1), minimal HE (n=1), and emerging therapies (n=2). Five observational cohort studies evaluated prognostic outcomes and biomarker-based risk stratification, including mortality and clinical outcomes (n=3) and biomarker prediction models (n=2). Because several studies addressed more than one clinical domain, some studies contributed to multiple sections of the narrative synthesis.

### Risk of bias assessment

Risk of bias assessment was performed using the Cochrane Risk of Bias 2.0 tool for randomized controlled trials and the Newcastle-Ottawa Scale (NOS) for observational studies. The results are summarized in Table 2, Figure 2, and Table 3.

**TABLE 2:** Risk of Bias Assessment of Randomized Controlled Trials Using the Cochrane RoB 2.0 Tool.

Study	Randomization	Missing Data	Outcome Measurement	Reporting	Overall
Bass 2010^9^	Low	Low	Low	Low	Low
Rahimi 2014^12^	Low	Low	Low	Low	Low
Sharma 2009^10^	Some concerns	Low	Some concerns	Low	Some concerns
Agrawal 2012^11^	Some concerns	Low	Some concerns	Low	Some concerns
Lunia 2014^14^	Some concerns	Low	Some concerns	Low	Some concerns
Sharma 2008^15^	Some concerns	Low	Some concerns	Low	Some concerns
Paik 2005^13^	Some concerns	Some concerns	Some concerns	Some concerns	Some concerns
Rockey 2014^16^	Low	Low	Low	Low	Low
Ventura-Cots 2021^18^	Low	Low	Low	Low	Low

RoB 2.0: Cochrane Risk of Bias 2.0 tool. Low: low risk of bias. Some concerns: some concerns regarding risk of bias. High: high risk of bias. Overall risk was determined according to the highest level of concern identified across individual domains.

**FIGURE 2:**
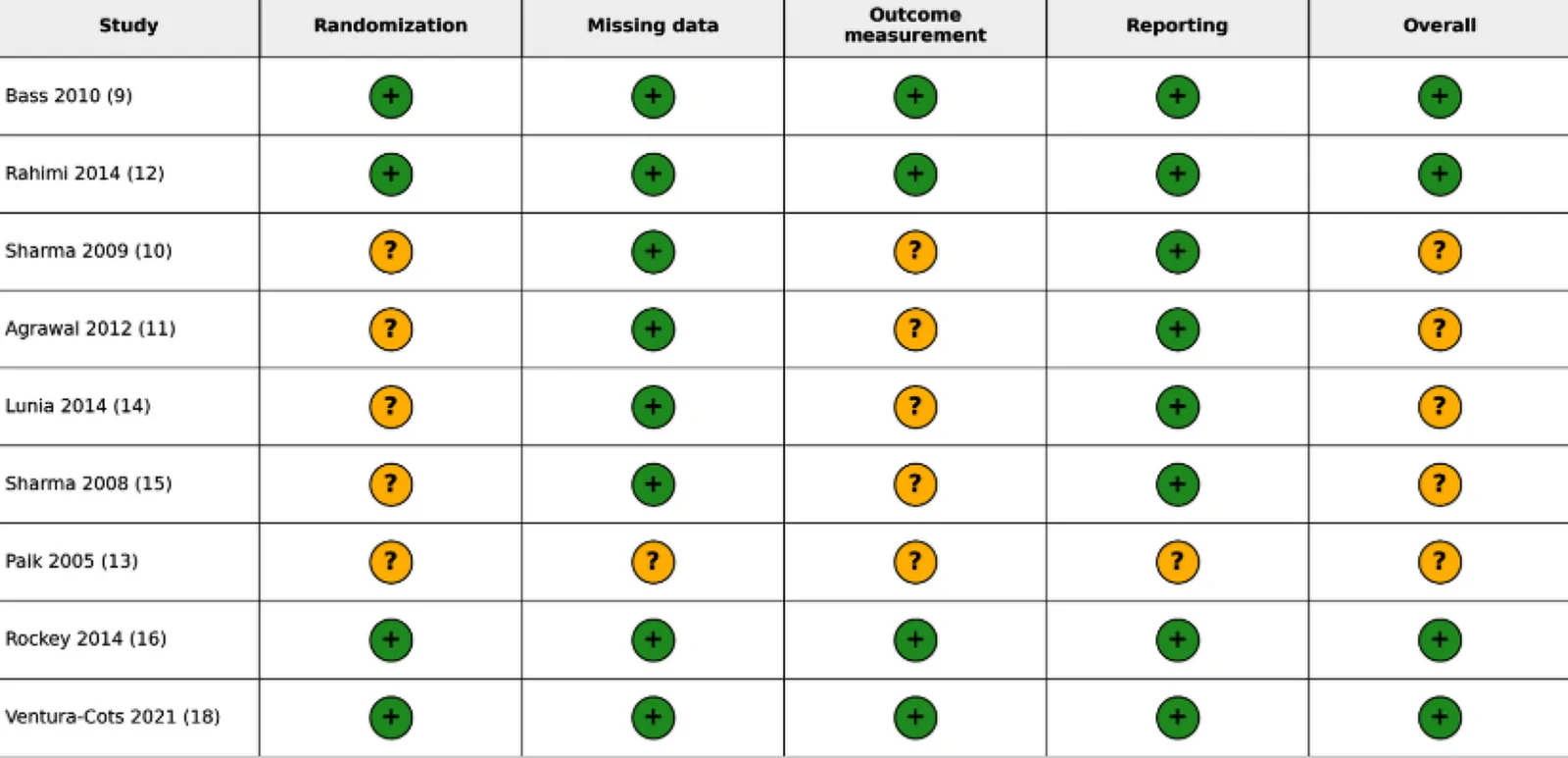
Risk of Bias Assessment of Randomized Controlled Trials Using the Cochrane RoB 2.0 Tool. RoB 2.0: Cochrane Risk of Bias 2.0 tool. Green circles indicate low risk of bias, yellow circles indicate some concerns, and red circles indicate high risk of bias. Domains evaluated included randomization process, missing outcome data, outcome measurement, and selective reporting. Overall risk of bias was assigned according to Cochrane RoB 2.0 recommendations based on the highest level of concern across domains.

**TABLE 3:** Methodological Quality Assessment of Observational Studies Using the Newcastle-Ottawa Scale (NOS).

Study	Selection	Comparability	Outcome	Total
Nardelli 2022^19^	4	2	3	9
Italian multicenter 2020^19^	4	2	3	9
Boeckmans 2025^21^	4	2	3	9
Elkrief 2023^22^	4	2	3	9
Sahney 2022^20^	3	2	2	7

NOS: Newcastle-Ottawa Scale. **NOS domains:** selection (maximum 4 points), comparability (maximum 2 points), and outcome (maximum 3 points). Total scores range from 0 to 9 points, with higher scores indicating better methodological quality.**Interpretation:** 7-9 points = high quality/low risk of bias; 5-6 points = moderate quality/moderate risk of bias; <5 points = low quality/high risk of bias.

Among the nine randomized controlled trials, overall methodological quality was moderate to high (Table 2, Figure 2). Four studies were judged as having low overall risk of bias, whereas five were classified as having some concerns, primarily related to open-label designs, incomplete reporting of allocation concealment, and potential deviations from intended interventions. No randomized trial was considered to be at high overall risk of bias.

Among the five observational studies, methodological quality was high according to the Newcastle-Ottawa Scale, with scores ranging from 8 to 9 points (Table 3). The main limitations identified were potential residual confounding, heterogeneity in the assessment of alcohol exposure, and variability in the definition and ascertainment of HE outcomes. Nevertheless, selection procedures, comparability of study groups, and outcome assessment were generally considered robust across studies.

Overall, interpretation of the available evidence should consider the substantial indirectness of the literature, as many studies were conducted in mixed-etiology cirrhotic populations rather than exclusively in patients with alcohol-related cirrhosis. This limitation was considered during the assessment of the certainty of evidence and should be taken into account when interpreting the findings.

### Certainty of evidence

The certainty of evidence according to the GRADE approach is summarized in Table 4. Evidence for secondary prophylaxis with rifaximin and lactulose was rated as moderate certainty, mainly because of indirectness related to the inclusion of mixed-etiology cirrhotic populations and limited alcohol-specific subgroup analyses. Evidence for polyethylene glycol in acute HE was rated as low certainty due to indirectness, imprecision, and the limited number of studies. Evidence regarding mortality and prognostic outcomes was rated as low certainty because it was derived primarily from observational cohorts and was limited by residual confounding and heterogeneity in alcohol exposure assessment. Evidence for biomarker-based risk prediction was rated as very low certainty due to indirectness, imprecision, and lack of external validation.

**TABLE 4:** GRADE Summary of Certainty of Evidence for Main Outcomes.

Outcome	Evidence base	Main limitations	Certainty of evidence
HE recurrence with rifaximin	1 randomized controlled trial	Indirectness due to mixed-etiology population; concomitant lactulose use; limited alcohol-specific subgroup analysis	Moderate
HE recurrence with lactulose	2 randomized controlled trials	Indirectness due to incomplete alcohol-specific stratification; open-label design in some studies	Moderate
Acute HE resolution with polyethylene glycol	1 randomized controlled trial	Indirectness, imprecision, single-center design, limited sample size	Low
Mortality and prognostic outcomes	Observational cohort studies	Residual confounding, heterogeneity in alcohol exposure assessment, variable HE definitions	Low
Biomarker-based risk prediction	Observational cohort studies	Indirectness, imprecision, limited validation, uncertain applicability to HE-specific outcomes	Very low

GRADE: grading of recommendations assessment, development and evaluation. HE: hepatic encephalopathy. Certainty levels were defined as high, moderate, low, or very low according to the GRADE framework. Downgrading was primarily based on indirectness, inconsistency, and imprecision.

### Secondary prophylaxis of hepatic encephalopathy

Evidence from four randomized controlled trials^9^ ^-^ ^11^ ^,^ ^13^ evaluated secondary prophylaxis of HE and suggests that rifaximin and lactulose may reduce recurrence rates. In a landmark multicenter randomized trial, rifaximin (550 mg twice daily) significantly reduced breakthrough HE episodes compared with placebo (HR 0.42; 95%CI 0.28-0.64), with recurrence rates at 6 months of 22.1% vs 45.9%, respectively. Notably, more than 90% of patients were receiving concomitant lactulose, supporting rifaximin as an adjunctive rather than monotherapy strategy^9^.

Similarly, lactulose demonstrated a robust effect in secondary prophylaxis. In a randomized trial with a median follow-up of 14 months, recurrence of overt HE was significantly lower in the lactulose group (19.6%) compared with placebo (46.8%; *P*=0.001), with persistent psychometric impairment emerging as an independent predictor of recurrence^10^. Comparative evidence suggests that both lactulose and probiotics are superior to no treatment in preventing recurrence, although differences between active treatments remain less clearly defined^11^.

### Acute management of hepatic encephalopathy

Two randomized controlled trials^12^ ^,^ ^13^ evaluated acute treatment strategies for HE. In the acute setting, polyethylene glycol (PEG) demonstrated superior efficacy compared with lactulose in hospitalized patients with overt HE. In the HELP trial, rapid catharsis with PEG resulted in a significantly higher proportion of patients achieving clinical improvement at 24 hours (91% vs 52%; *P*<0.01), greater reduction in HE severity scores, and shorter time to resolution^12^. However, the relatively small sample size and single-center design limit generalizability.

Comparative data between rifaximin and lactulose in acute HE remain limited, with incomplete reporting in available studies precluding quantitative synthesis^13^.

### Primary prophylaxis and minimal hepatic encephalopathy

Two randomized controlled trials^14^ ^,^ ^15^ evaluated primary prophylaxis and minimal HE. Probiotics showed efficacy in primary prophylaxis of HE. In patients without prior overt HE, probiotic therapy significantly reduced the incidence of overt HE and was associated with improvements in ammonia levels, gut microbiota parameters, and neurocognitive performance^14^.

In minimal HE, lactulose, probiotics, and combination therapy demonstrated comparable efficacy in improving psychometric performance and neurophysiological markers, without statistically significant differences between strategies^15^.

### Emerging and adjunctive therapies

Three randomized controlled trials^16^ ^-^ ^18^ evaluated emerging or adjunctive therapies. Emerging therapeutic approaches demonstrated signal-level efficacy but remain investigational. Glycerol phenylbutyrate (GPB), a nitrogen-scavenging agent, significantly reduced HE events and related hospitalizations in a phase II trial^16^. Fecal microbiota transplantation (FMT), evaluated in a pilot randomized study, was associated with improvements in cognition and microbiome composition; however, the small sample size and limited safety data preclude clinical recommendations^17^.

Intravenous albumin did not demonstrate a statistically significant benefit in transplant-free survival at 90 days in patients with overt HE, although the study was likely underpowered to detect clinically meaningful differences^18^.

### Prognostic factors and clinical outcomes

Three observational cohort studies^19^ ^-^ ^21^ evaluated mortality and clinical outcomes. Observational studies consistently identified HE as a major prognostic determinant in alcohol-related cirrhosis. In a large retrospective cohort of 1,429 hospitalizations, HE was present in 18.5% of admissions and independently associated with increased 90-day mortality (OR 2.84), along with acute kidney injury, gastrointestinal bleeding, and infection^19^.

Similarly, in a prospective multicenter cohort, HE at admission was associated with significantly increased mortality (HR 3.1 at 90 days; HR 2.9 at 1 year). Importantly, active alcohol consumption emerged as an independent predictor of mortality (HR 2.5), underscoring its role as a key disease modifier^20^.

Registry-based data further demonstrated that while the risk of hepatic decompensation was comparable between alcohol-related and metabolic etiologies, post-event mortality was lower in al­cohol-related cirrhosis, potentially reflecting the impact of alcohol abstinence as a modifiable factor^21^.

### Biomarkers and risk stratification

One observational cohort study^22^ evaluated biomarker-based risk stratification. Biomarker-based risk stratification showed promising results in compensated alcohol-related cirrhosis. The combination of cytokeratin-18 (K18) and fibrosis markers identified patients at increased risk of liver-related events. In individuals with active alcohol consumption, liver-derived extracellular vesicles (lEV) further improved risk discrimination, identifying a subgroup with markedly elevated event rates^22^.

## DISCUSSION

This systematic review synthesizes the available evidence on HE in alcohol-related cirrhosis, integrating data from 14 studies encompassing 6,185 participants across acute management, primary and secondary prophylaxis, and prognostic assessment. The available evidence suggests that HE occurring in patients with alcohol-related cirrhosis may be associated with adverse clinical outcomes, including 90-day mortality exceeding 12% in some hospitalized cohorts and an increased risk of short-term mortality among patients presenting with HE^19^ ^,^ ^20^. Several studies reported an association between HE at admission and subsequent mortality, with hazard ratios reaching 3.1, although interpretation should be tempered by the observational nature of much of the available evidence^19^ ^,^ ^20^. Furthermore, active alcohol consumption was identified as an independent predictor of mortality in one prospective cohort study (HR 2.5), supporting the hypothesis that ongoing alcohol exposure may contribute to poorer outcomes and highlighting the potential importance of integrating alcohol use disorder management into the overall care of these patients^20^.

From a therapeutic perspective, rifaximin appears to be the intervention supported by the strongest available evidence for secondary prophylaxis of hepatic encephalopathy. The available data suggest a reduction in breakthrough HE events, with the pivotal randomized trial reporting a hazard ratio of 0.42 for recurrence compared with placebo^9^. However, interpretation of the independent effect of rifaximin should be undertaken cautiously, given the near-universal concomitant use of lactulose in that study, which reflects routine clinical practice but limits attribution of treatment effect to rifaximin alone.

Similarly, lactulose remains a widely used strategy for secondary prophylaxis. Randomized studies suggest a clinically meaningful reduction in recurrence compared with placebo^10^, although the certainty of evidence is tempered by study heterogeneity and the limited availability of alcohol-specific analyses. Taken together, the current literature supports the use of both rifaximin and lactulose for recurrence prevention, while highlighting the need for studies specifically focused on patients with alcohol-related cirrhosis^9^ ^,^ ^10^.

Comparative data between rifaximin and lactulose remain limited, as available trials do not provide sufficient detail to permit robust quantitative synthesis or definitive conclusions regarding comparative effectiveness^13^. In the acute inpatient setting, the available evidence suggests that polyethylene glycol (PEG) may be associated with more rapid resolution of HE compared with lactulose, including higher rates of clinical improvement at 24 hours and shorter time to symptom resolution^16^. However, these findings are derived from a limited number of studies with relatively small sample sizes and restricted alcohol-specific analyses. Consequently, the certainty of evidence remains low, and the applicability of these results to patients with alcohol-related cirrhosis should be interpreted cautiously

Emerging therapeutic approaches remain promising but should currently be considered investigational. The available evidence suggests that nitrogen-scavenging agents such as glycerol phenylbutyrate (GPB) may be associated with reductions in HE events and HE-related hospitalizations^16^. Similarly, microbiome-based interventions, including fecal microbiota transplantation, have been associated with improvements in cognitive outcomes and microbiome composition in preliminary studies; however, the available evidence remains limited by small sample sizes and the exploratory nature of the published data^17^. Intravenous albumin has been evaluated as an adjunctive therapy in overt HE, but current studies have not consistently shown a significant survival benefit, and available results remain limited by imprecision and insufficient statistical power^18^. Consequently, further adequately powered studies are required before these interventions can be routinely recommended in clinical practice.

The available evidence raises the possibility that HE occurring in alcohol-related cirrhosis may involve pathophysiological mechanisms extending beyond classical hyperammonemia. Experimental and translational studies have suggested that alcohol exposure may contribute to neurotoxicity through multiple pathways, including acetaldehyde-mediated neuronal dysfunction, alterations in GABAergic and glutamatergic neurotransmission, gut dysbiosis, endotoxemia, and systemic inflammation^7^ ^,^ ^8^. The interaction between hyperammonemia, neuroinflammation, and alcohol-related neurobiological alterations provides a biologically plausible explanation for potential differences in clinical presentation and outcomes. However, the current literature does not allow definitive confirmation that these mechanisms translate into a distinct HE phenotype in patients with alcohol-related cirrhosis, and further prospective studies are required to clarify this hypothesis^7^ ^,^ ^8^.

Biomarker-based risk stratification represents an emerging area of interest in alcohol-related cirrhosis. The available evidence suggests that hepatocyte-derived markers such as cytokeratin-18 (K18) and liver-derived extracellular vesicles (lEV) may help identify patients at increased risk of liver-related events, including those with compensated disease^22^. Furthermore, integration of these biomarkers with conventional fibrosis indices has been associated with improved risk discrimination in some cohorts, particularly among patients with ongoing alcohol consumption^22^. However, these findings are derived from a limited number of observational studies and require external validation before they can be incorporated into routine clinical practice. Consequently, the role of these biomarkers should currently be considered promising but exploratory^22^.

Registry-based data suggest that the risk of hepatic decompensation may be broadly comparable between alcohol-related and metabolic cirrhosis, whereas post-event mortality may differ between these etiologies^21^. The mechanisms underlying these observations remain uncertain and may be influenced by differences in patient characteristics, disease progression, access to care, and patterns of alcohol consumption. One potential explanation is the possibility that sustained alcohol abstinence modifies the natural history of alcohol-related liver disease, although this hypothesis has not been directly evaluated in the studies included in this review^21^. These findings highlight the importance of assessing and managing alcohol use disorder as part of the comprehensive care of patients with alcohol-related cirrhosis and hepatic encephalopathy. However, the available evidence does not allow definitive conclusions regarding the specific impact of individual pharmacological interventions, and further prospective studies are needed to clarify their effect on HE-related outcomes.

To facilitate interpretation of the available evidence, Figure 3 summarizes the proposed evidence-based clinical framework for hepatic encephalopathy in alcohol-related cirrhosis. The model integrates the principal findings identified in this review, highlighting the role of active alcohol consumption as a prognostic modifier, the evidence supporting lactulose and rifaximin for secondary prophylaxis, the potential utility of polyethylene glycol during acute episodes, and the emerging role of biomarker-based risk stratification. Importantly, the framework also emphasizes the substantial indirectness of the current literature and the need for dedicated prospective studies specifically focused on alcohol-related cirrhosis (Figure 3).

**FIGURE 3:**
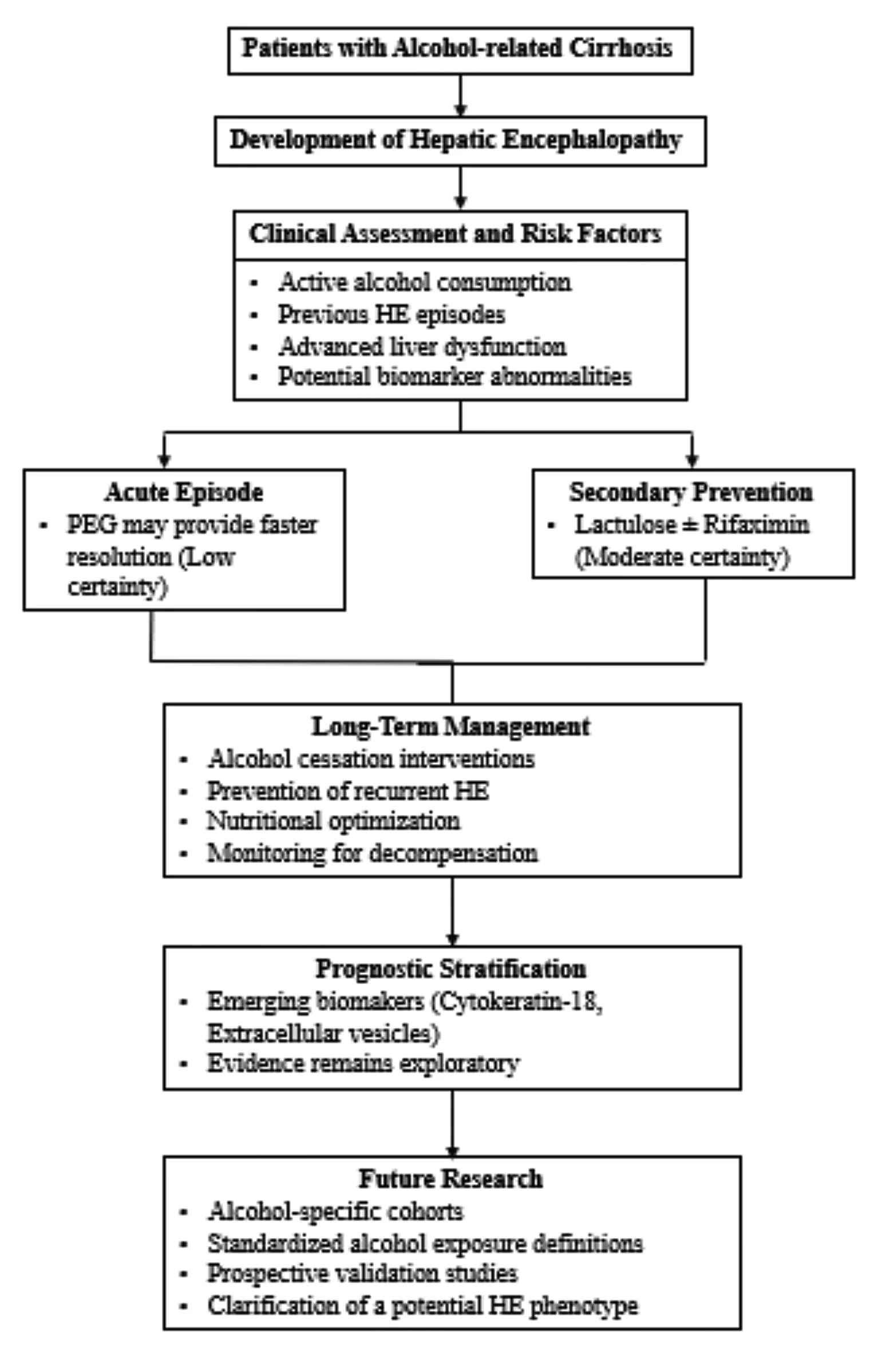
Evidence-Based Clinical Framework for Hepatic Encephalopathy in Alcohol-Related Cirrhosis. Evidence-based clinical implications and management considerations for hepatic encephalopathy in alcohol-related cirrhosis. Current evidence suggests that recurrence prevention with lactulose and rifaximin is supported by the strongest available data, whereas polyethylene glycol may accelerate resolution of acute episodes. Active alcohol consumption appears to be associated with adverse outcomes and should be systematically assessed. Biomarker-based risk stratification remains investigational. The substantial indirectness and heterogeneity of the available literature highlight the need for dedicated prospective studies in alcohol-related cirrhosis.

The present review highlights several important knowledge gaps. First, the field lacks prospective cohorts specifically designed to evaluate HE in alcohol-related cirrhosis. Most available studies include mixed-etiology cirrhotic populations, limiting alcohol-specific inference. Future studies should enroll predefined alcohol-related cirrhosis cohorts and report outcomes separately according to liver disease etiology. Second, alcohol exposure remains inconsistently defined across studies. Some reports classify patients by historical etiology, whereas others evaluate active alcohol use, without standardized quantification of intake, duration of abstinence, relapse, or alcohol use disorder severity. Future research should incorporate standardized alcohol exposure definitions, validated screening tools, longitudinal alcohol-use assessment, and clear distinction between past alcohol-related liver disease and ongoing alcohol consumption. Third, the interaction between alcohol exposure, systemic inflammation, gut dysbiosis, and hyperammonemia remains insufficiently characterized in clinical studies. Translational cohorts integrating neurocognitive testing, inflammatory markers, ammonia levels, microbiome profiling, and alcohol-use trajectories could clarify whether alcohol-related HE represents a distinct biological phenotype or a variation within conventional cirrhosis-related HE. Fourth, therapeutic evidence remains largely extrapolated from mixed-etiology trials. Dedicated randomized controlled trials or adequately powered subgroup analyses are needed to determine whether treatment response to lactulose, rifaximin, polyethylene glycol, microbiome-based interventions, or alcohol-use-disorder therapies differs in alcohol-related cirrhosis. Finally, prognostic models and biomarkers require external validation. Future studies should evaluate whether biomarkers such as cytokeratin-18, extracellular vesicles, fibrosis indices, and alcohol-use metrics improve prediction of HE recurrence, hospitalization, mortality, or transplant-free survival beyond conventional clinical scores.

This review has several strengths. To our knowledge, it represents the first systematic synthesis specifically focused on hepatic encephalopathy in alcohol-related cirrhosis, integrating evidence from randomized controlled trials and observational studies across therapeutic, prognostic, and biomarker-related domains. Methodological rigor was enhanced through systematic literature searches across multiple databases, formal risk-of-bias assessment, and evaluation of certainty of evidence using the GRADE framework. Nevertheless, several limitations should be acknowledged. First, the review protocol was not prospectively registered in PROSPERO or another international registry for systematic reviews. Consequently, the review may be more susceptible to reporting bias than prospectively registered reviews. Nevertheless, the review methodology, eligibility criteria, search strategy, study selection process, risk-of-bias assessment, and certainty-of-evidence evaluation were predefined before data synthesis and are fully described in the manuscript to enhance transparency and reproducibility. Second, substantial clinical and methodological heterogeneity was identified across studies, including variability in patient populations, definitions of hepatic encephalopathy, assessment of alcohol exposure, interventions, and outcome measures. Consequently, quantitative meta-analysis was not considered appropriate and a narrative synthesis was performed instead. Third, most included studies were not specifically designed to evaluate hepatic encephalopathy in alcohol-related cirrhosis as a distinct clinical entity. As a result, much of the available evidence was derived from mixed-etiology cirrhotic populations, introducing a significant degree of indirectness and limiting the certainty of conclusions applicable specifically to alcohol-related cirrhosis. Fourth, risk-of-bias assessment identified methodological limitations in several studies, including open-label trial designs, incomplete reporting of allocation concealment, potential performance bias, residual confounding, and heterogeneity in alcohol exposure assessment. Consistent with these findings, GRADE evaluation indicated low-to-moderate certainty for most clinically relevant outcomes, largely because of indirectness, inconsistency, and imprecision.

Future research should prioritize prospective studies specifically designed for patients with alcohol-related cirrhosis, incorporating standardized definitions of alcohol exposure, uniform diagnostic criteria for hepatic encephalopathy, and alcohol-specific outcome reporting. Such studies will be necessary to clarify optimal therapeutic strategies, validate emerging biomarkers and interventions, and determine whether the observed clinical differences truly represent a distinct HE phenotype or reflect variations within the broader spectrum of cirrhosis-related HE.

An important unresolved question is whether hepatic encephalopathy occurring in alcohol-related cirrhosis represents a distinct clinical phenotype or simply a variation within the broader spectrum of cirrhosis-related HE. Although experimental and observational studies suggest potential biological and prognostic differences, the current evidence base remains insufficient to establish this concept definitively. Dedicated prospective studies specifically designed for alcohol-related cirrhosis will be required to determine whether such a phenotype exists and whether it has meaningful implications for diagnosis, prognosis, or therapeutic decision-making.

## CONCLUSION

The available evidence suggests that HE occurring in patients with alcohol-related cirrhosis is associated with adverse clinical outcomes, including increased short-term mortality and recurrent hepatic decompensation. However, much of the current literature is derived from mixed-etiology cirrhotic populations, limiting the certainty of conclusions that can be drawn specifically for alcohol-related cirrhosis.

Current evidence supports the use of rifaximin and lactulose for the prevention of recurrent hepatic encephalopathy, while polyethylene glycol may be associated with more rapid resolution of acute episodes, although the certainty of evidence remains limited. Emerging therapies and biomarker-based risk stratification strategies appear promising but require further validation in dedicated alcohol-related cirrhosis cohorts.

Overall, the available literature supports the hypothesis that HE in alcohol-related cirrhosis may exhibit particular clinical and prognostic characteristics. Nevertheless, the existence of a distinct alcohol-related HE phenotype cannot be definitively established based on current evidence and requires confirmation through well-designed prospective studies. Future research should prioritize alcohol-specific cohorts with standardized exposure definitions and outcome reporting to improve the understanding and management of this complex condition.

## Data Availability

not applicable - the study did not use research data
